# The Use of Fluorescence Microscopy to Study the Association Between Herpesviruses and Intrinsic Resistance Factors

**DOI:** 10.3390/v3122412

**Published:** 2011-12-07

**Authors:** Roger D. Everett

**Affiliations:** The Medical Research Council (MRC) - University of Glasgow Centre for Virus Research, 8, Church Street, Glasgow G11 5JR, Scotland, UK; Email: roger.everett@glasgow.ac.uk; Tel.: +44-141-330-3923; Fax: +44-1411-330-3520

**Keywords:** Herpes Simplex Virus type 1, ICP0, intrinsic antiviral resistance, ND10, PML nuclear bodies, SUMO

## Abstract

Intrinsic antiviral resistance is a branch of antiviral defence that involves constitutively expressed cellular proteins that act within individual infected cells. In recent years it has been discovered that components of cellular nuclear structures known as ND10 or PML nuclear bodies contribute to intrinsic resistance against a variety of viruses, notably of the herpesvirus family. Several ND10 components are rapidly recruited to sites that are closely associated with herpes simplex virus type 1 (HSV-1) genomes during the earliest stages of infection, and this property correlates with the efficiency of ND10 mediated restriction of HSV-1 replication. Similar but distinct recruitment of certain DNA damage response proteins also occurs during infection. These recruitment events are inhibited in a normal wild type HSV-1 infection by the viral regulatory protein ICP0. HSV‑1 mutants that do not express ICP0 are highly susceptible to repression through intrinsic resistance factors, but they replicate more efficiently in cells depleted of certain ND10 proteins or in which ND10 component recruitment is inefficient. This article presents the background to this recruitment phenomenon and summaries how it is conveniently studied by fluorescence microscopy.

## 1. Introduction

Intrinsic antiviral resistance (also known as intrinsic immunity) is a relatively recently described antiviral defence. Unlike acquired immunity and cytokine mediated innate immunity, intrinsic resistance functions through constitutively expressed cellular proteins that restrict virus replication within an individual infected cell [1,2]. Intrinsic resistance involves diverse proteins and mechanisms, depending on the particular virus in question. This short review will focus on herpesviruses, and in particular on the visualization of the behaviour of intrinsic resistance factors by fluorescence microscopy. The emphasis will be on herpes simplex virus type 1 (HSV-1), but it is likely that much of what has been found in HSV-1 infected cells also occurs during other herpesvirus infections, and may also extend to other nuclear replicating DNA viruses.

## 2. Interactions Between DNA Viruses and ND10

The history of the visualization of intrinsic resistance factors during herpesvirus infection dates back to early studies by Gerd Maul and colleagues, who discovered that a group of then uncharacterized cellular proteins form distinct nuclear foci. He named these structures ND10 (Nuclear Domain 10), representing their approximate average number per cell, and found that they were disrupted during stress and HSV-1 infection [3]. Later it was found that their core component is the promyelocytic leukemia protein (PML), so they are also commonly known as PML nuclear bodies (PML NBs). The disruption of ND10 during HSV-1 infection is caused by the viral regulatory protein ICP0, a RING finger ubiquitin ligase that induces the degradation of PML [4,5]. Although disruption or alteration of ND10 proved to be a common feature of many DNA virus infections (reviewed in [6,7]), for many years it was unclear whether ND10 were beneficial or inhibitory for virus infection. More recently, the use of RNA interference has provided increasing evidence that ND10 components including PML, Sp100, hDaxx and ATRX have a restrictive effects on both HSV-1 and human cytomegalovirus (HCMV) [8,9,10,11,12,13,14,15,16,17,18,19]. These inhibitory effects are overcome by viral regulatory proteins, ICP0 in the case of HSV-1, and the combination of pp71 and IE1 (IE72) of HCMV (see references cited above, and references therein). Virus mutants with lesions in any of these three proteins have a low probability of initiating a productive infection because viral transcription is repressed. Although the reasons for this repression have not been fully explained, and are in some cases controversial, ICP0 null mutant HSV-1 has an increased probability of initiating a lytic infection in cells depleted of PML, Sp100, hDaxx or ATRX [9,10,12], HCMV replicates more efficiently in PML-, hDaxx- or Sp100‑depleted cells [15,17,19,20], and pp71 HCMV mutants are at least partially complemented in hDaxx and ATRX depleted cells [8,13,14]. Therefore these prominent ND10 components have properties consistent with the definition of cellular intrinsic resistance factors. 

## 3. Association of Herpesvirus Genomes with ND10

The interactions between herpesviruses and ND10-related intrinsic resistance factors are particularly amenable to study by microscopy, not only because ND10 are disrupted during infection, but also because viral genomes become associated with ND10 proteins. This was first observed with ICP0 null mutant HSV-1 [21], and this was extended to include an association between ND10 and the genomes and/or replication compartments of several nuclear replicating DNA viruses [22,23]. The core of this article describes how visualization of this association has enabled progress on understanding its mechanism and functional consequences. Note that a distinction is being made between recruitment of cellular proteins into viral replication compartments themselves [24,25,26] and the recruitment of proteins to sites that are juxtaposed to, overlapping or associated with viral genome foci, rather than being closely co-localized.

## 4. The Recruitment of ND10 Proteins to Novel ND10-Like Foci Associated with Viral Genomes

**Figure 1:**
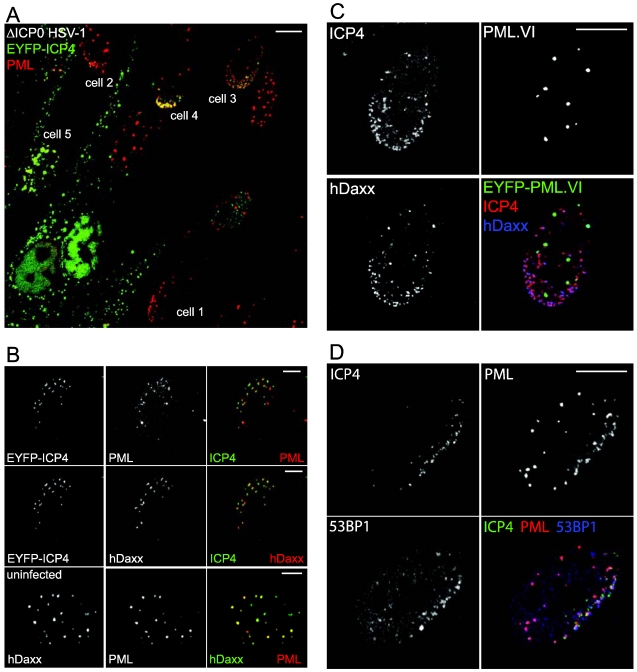
Examples of recruitment of ND10 components to sites associated with HSV-1 genomes. (**A**) A view of an edge of a developing plaque. The centre of the plaque is down and left of the lower left corner of the image. The cells were infected with an ICP0‑deficient virus expressing EYFP-linked ICP4 (green) then developing plaques were examined 24 h later. PML was detected using anti-PML monoclonal antibody 5E10 (red). The numbered cells show different stages of recruitment as infection develops: cell 1—asymmetric distribution of PML can occur before a cell has detectable ICP4 expression (compare with the random distribution of PML foci in the cell to the right of cell 3 and in the bottom row of panel 3); cell 2—asymmetric distribution of PML with very small ICP4 foci; cell 3—multiple small ICP4 and PML foci; cell 4—larger ICP4 foci, with overlapping strong PML foci; cell 5—a cell in the mid stages of infection with developing replication compartments associated with small remaining foci of PML. (**B**) More detailed examples of ICP4 association with PML (top row), hDaxx (in the same cell, middle row) in comparison with an uninfected cell (bottom row). (**C**) Use of a cell line depleted for endogenous PML and reconstructed with expression of EYFP-linked PML.VI (green), an isoform that lacks the SIM. The cells were infected with ICP0 null mutant HSV-1 and stained for ICP4 expression (monoclonal antibody 58S, red) and hDaxx (rabbit polyclonal antibody from Upstate, blue). Note efficient recruitment of hDaxx, despite the lack of any recruitment of PML.VI. (**D**) Recruitment of the DNA damage response protein 53BP1 (blue) to regions in close proximity to ICP4 foci (EYFP-ICP4, green in cells infected with ICP0 null mutant virus expressing EYFP-linked ICP4. PML (red) is recruited to ICP4 associated foci that are distinct from those of 53BP1. The scale bars show 10 μm in A, C and D, 5 μm in B.

The association of viral genomes with ND10 poses the question whether it is the viral genomes or pre-existing ND10 structures that move through the nucleoplasm until they encounter each other. In fact, neither of these alternatives is correct. In an entirely serendipitous discovery, it was found that novel ND10-like structures formed *de novo* at the sites of the viral genomes through the recruitment of ND10 component proteins. Cells surrounding developing plaques frequently display foci of the HSV-1 transcriptional regulator ICP4 in an asymmetric pattern close to the inner side of the nuclear envelope [27] (Figure 1). This is most marked when the foci are small, indicating an early stage of infection. ICP4 serves as a marker for viral genomes and replication compartments because it binds avidly to multiple sites throughout the viral genome [28,29,30,31]. These asymmetric ICP4 foci are associated with or juxtaposed to accumulations of ND10 proteins, indicating that the association of HSV-1 genomes and ND10 occurs through recruitment of ND10 components [32] (Figure 1B). 

This asymmetric pattern of ICP4 foci can be explained by two factors. Firstly, HSV-1 spreads via cell to cell contact as a plaque expands, therefore the virus invades newly infected cells preferentially from one direction. In addition, HSV-1 capsids are carried on microtubules to the nuclear envelope, via the microtubule organising centre (MTOC). The capsids then associate with nuclear pores in the vicinity of the MTOC [33,34]. The viral genomes are therefore released into the nucleoplasm in an asymmetric manner, and the pattern of ICP4 foci in cells at the edge of a developing plaque reflects this. On the other hand, when virus is added to the surface of a cell monolayer in a normal high multiplicity infection, virus particles may reach the nuclear envelope more directly, giving a more random distribution of viral genomes in the nucleus. This would explain the interpretation of the initial studies that the viral genomes move through the nucleoplasm until they encounter a pre-existing ND10 structure. Live cell microscopy demonstrated that the association between viral genomes and ND10 proteins results from the deposition of ND10 components in new, ND10-like structures, rather than the movement of intact, pre-existing ND10 [32].

This phenotype is particularly easy to detect in developing HSV-1 plaques for several reasons. HSV-1 replicates very efficiently, producing in a relatively short space of time a large number of progeny particles. Therefore there is a rapid asymmetric delivery of a large number of genomes into the nucleus, each of which results in the recruitment of ND10 proteins, making the phenomenon obvious. If there were fewer genomes entering the nucleus it would be much more difficult to determine whether the genomes were associated with pre-existing or newly formed ND10-like structures. It is important that the effect was observed first using an ICP0-null mutant virus. During a normal infection, ICP0 very rapidly results in the disruption of ND10 [3]. Therefore, although the recruitment occurs in a wild type virus infection, it is short lived and thus difficult to detect [32].

These factors may explain why the phenomenon has not yet been studied in other virus infections. Given what is known about the mechanism (see below), it is likely that similar recruitment also occurs to the parental genomes of other viruses with large DNA genomes. However, one or more of the above criteria may be less favourable in other cases. For example, HCMV replicates much more slowly than HSV-1, which likely results in a less synchronous and lower dose delivery of progeny particles to cells in a developing plaque. Furthermore, HCMV mutants lacking the regulatory proteins that might be expected to inhibit recruitment (pp71 and IE1) are very difficult to propagate, thereby making studies on their plaque development more challenging than analogous studies on ICP0 null mutant HSV-1. Although ICP0 HSV-1 mutants have a greatly reduced ability to initiate a productive infection, once a cell becomes productively infected a similar amount of progeny virus is released to that in a wild type virus infection, and the resulting high dose of virus delivered to neighbouring cells ensures that the defect due to the lack of ICP0 is overcome [35].

## 5. Experimental Approaches

Observation of the recruitment of ND10 components to sites associated with viral genomes requires a suitable infection regime and reagents for detecting viral DNA and the recruited proteins of interest. Although preferable, it is not absolutely essential to examine a developing plaque because nuclear asymmetry of HSV-1 genomes can be provided by the concentrating effect of the MTOC (see above). Therefore, particularly in thinly seeded cells with a large cytoplasmic area such as human fibroblasts (HFs), a proportion of cells infected at high multiplicity in a conventional manner will exhibit multiple asymmetric ICP4 foci. The presence of asymmetric viral genome foci is important because without them it is more difficult to exclude the possibility of chance association with ND10 rather than recruitment.

The major transcriptional regulator ICP4 provides a convenient marker for HSV-1 genomes because it is expressed at immediate-early times and binds to both parental genomes and developing replication compartments [28,29,30,31]. Although viral DNA replication proteins such as ICP8 also localise to replication compartments [36], they are not as useful for these studies because they are not expressed during the earliest stages of infection when the recruitment of cellular proteins is most obvious. Viral genomes can also be detected by fluorescence *in situ* hybridisation [32]. Although this was the method by which viral genome association with ND10 was first studied [21], it is technically much more challenging and may not always be compatible with some antibodies used to detect recruited proteins. A powerful variation is the use of autofluorescent viral or cellular fusion proteins [32,37,38]. These systems increase overall flexibility and allow straightforward triple label experiments. 

An approach that has proved very productive combines lentivirus mediated stable depletion of a cellular protein of interest using RNA interference, followed by reconstituted expression of a tagged version of the same protein using a second lentiviral vector. This allows the study of mutant forms of a protein in the absence of the potentially complicating factor of the wild type version [12,38,39] (see Figure 1C). It is preferable to use a weak promoter in the vector to avoid any non-physiological consequences of over-expression of the protein of interest.

## 6. Factors Involved in ND10 Recruitment

Recruitment of ND10 components to sites associated with HSV-1 genomes is not dependent on viral transcription [32] and it can occur very rapidly, within minutes of virus being added to the cell monolayer [40] and in some cells before expression of ICP4 is readily detectable (Figure 1A). Therefore it appears that the cell is responding to the entry of the viral genome into the nucleus. Given that PML is required for the ND10 formation in uninfected cells [41,42], it is surprising that the recruitment of other ND10 components, such as Sp100, hDaxx and ATRX occurs in a PML‑independent manner [9,12]. Therefore formation of normal ND10 and virus-induced ND10-like structures occur by distinct mechanisms. Similarly recruitment of hDaxx and PML are not dependent on Sp100 [10], and recruitment of Sp100 and PML does not require hDaxx [43]. In contrast, recruitment of ATRX is dependent on its ability to interact with hDaxx [12]. While several major ND10 components can be recruited independently of each other, it seemed likely some common or related mechanism might be involved. A comparison of the recruitment potential of different PML isoforms demonstrated that the presence of a SUMO interaction motif (SIM) is required, and this also proved to be the case for Sp100 and hDaxx [38]. Figure 1C shows an example of lack of recruitment a SIM-deficient PML isoform, while hDaxx in the same cell is recruited normally. While SUMO modification of PML is also required for its recruitment, this was not the case for Sp100 [38]. Taken with the fact that hDaxx is not SUMO modified to any significant extent in normal circumstances, it appears that the SIM rather than SUMO modification *in cis* is the important common factor between these three recruited proteins. These studies were made possible by the depletion/reconstruction methodology noted above because the presence of endogenous PML or Sp100 has a dominant effect on the behaviour of introduced mutant forms of these proteins, thereby masking the effect of the SIM [38]. 

The requirement for their SIMs for PML and Sp100 recruitment implies that these proteins are responding to some other SUMO modification event. Consistent with this hypothesis, SUMO conjugates and a SUMO E3 ligase (PIAS2β) were also found in the recruited foci, in a PML independent manner [38]. Furthermore, recruitment was greatly reduced in cells depleted of Ubc9, the sole SUMO E2 conjugating enzyme [44]. It seems that the entry of the HSV-1 genome into the nucleus triggers SUMO conjugation events that lead to a cascade of protein recruitment, at least some of which takes place in a SUMO interaction dependent manner. A catalogue of proteins that are known to be recruited to such HSV-1 genome associated sites is presented in Table 1.

**Table 1:** Cellular proteins recruited to HSV-1 associated foci.

Protein	Comments	Category	References
PML	SIM and SUMO modification dependent	ND10	[32,38]
Sp100	SIM dependent, not dependent on SUMO modification or PML	ND10	[32,38]
hDaxx	SIM dependent, independent of PML and ATRX	ND10	[12,32,38]
ATRX	Dependent on hDaxx	ND10	[12]
SUMO-1	Much more prominent in the presence than absence of PML	ND10/ protein modification	[38,44]
SUMO-2/3	PML independent	ND10/ protein modification	[38,44]
PIAS2β	Independent of PML in human fibroblasts	ND10	[38]
γ-H2AX	Does not overlap with ICP4 signal, ND10 independent, not inhibited by ICP0	DNA damage response	[45]
Mdc1	Does not overlap with ICP4 signal, ND10 independent, not inhibited by ICP0	DNA damage response	[45]
Brca1	Does not overlap with ICP4 signal, ND10 independent, inhibited by ICP0	DNA damage response	[45]
53BP1	Does not overlap with ICP4 signal, ND10 independent, dependent on RNF8 and RNF168, inhibited by ICP0	DNA damage response	[45]
Conjugated ubiquitin	Dependent on RNF8 and RNF168 in ΔICP0 infections	Protein modification	[45]

## 7. Biological Significance of ND10 Component Recruitment

Recruitment of cellular proteins to the sites of HSV-1 genomes could in principle have either positive or negative effects, depending on the protein in question. There are many cellular proteins that are required for efficient viral gene expression, components of the transcriptional apparatus, for example. However, it was not until the RNAi studies cited above that it became clearer that ND10 proteins had an inhibitory effect on HSV-1 replication that is normally counteracted by ICP0. The links between recruitment of these proteins to virus-induced ND10-like structures and intrinsic resistance have come from two directions. Firstly, there is a very good correlation between the abilities of different mutant forms of ICP0 to impede recruitment and to stimulate infection [46]. Secondly, while reconstituted expression of wild type forms of PML isoform I and hDaxx reverses the increase in ICP0 null mutant HSV-1 plaque formation that occurs in cells depleted of these proteins, recruitment‑defective versions of these proteins are unable to do so [12,39].

It is intriguing that repressed HSV-1 genomes in quiescently infected cells are enclosed within expanded ND10-like structures [40]. Viral genomes sequestered in this manner may be inaccessible to the transcriptional apparatus, and such sequestration may be the end point of the recruitment process if a genome fails to initiate an efficient infection. It may not be a coincidence that ICP0, which would disrupt such shell-like structures, is able to reactivate viral gene expression in such quiescently infected cells.

## 8. Recruitment of ND10 Components to Other DNA Virus Genomes

While it is clear that close association between ND10 and the genomes of a variety of DNA viruses is commonplace [21,22,23], whether this is due to recruitment of ND10 components or movement of the genomes to pre-existing ND10 has not been determined in viruses other than HSV-1. Given that recruitment to HSV-1 genomes does not require viral transcription or *de novo* protein synthesis (see above), the simplest hypothesis is that it is the nuclear entry of a large unchromatinized viral DNA molecule that triggers the response. Therefore it seems likely that similar events occur in infections by other viruses with similar DNA genomes and modes of nuclear entry, notably other members of the herpesvirus family. There is some evidence that is consistent with analogous recruitment occurring during HCMV infection. The viral regulatory protein IE2, which is functionally analogous to ICP4, forms foci during the early stages of infection that co-localise with viral genomes and are closely associated with ND10 [15,47]. While this does not necessarily reflect ND10 protein recruitment, IE2 also forms foci during infection of PML depleted cells, and this stimulates the transient appearance of closely associated accumulations of hDaxx and co-localizing Sp100 [15]. In uninfected PML depleted cells these two ND10 proteins are dispersed and do not form any co-localising foci, so their aggregation in association with IE2 foci during infection is entirely consistent with recruitment into novel foci. Similar recruitment of hDaxx and Sp100 into foci associated with those of ICP4 also occurs during HSV-1 infection of PML depleted cells [9].

In contrast, a plaque assay model of infection by varicella zoster virus (VZV) revealed no association between PML and foci of the orf62 protein (the VZV orthologue of ICP4) [48]. This was despite the presence of cells with asymmetrically distributed orf62 foci analogous to those of ICP4 in developing HSV-1 plaques. This could indicate that ND10 component recruitment does not occur in response to nuclear entry of VZV genomes, or that expression of orf61 (the ICP0 orthologue of VZV) inhibited the recruitment.

## 9. Recruitment of DNA Repair Proteins to Sites Associated with HSV-1 Genomes

An exciting recent extension to the recruitment studies involving ND10 proteins comes from the observation that certain DNA damage response proteins are also recruited to sites that are juxtaposed to HSV-1 genomes during the early stages of infection [45]. It has previously been established that HSV-1 induces a DNA damage response, and that a consequence of ICP0 expression is to disrupt the formation of irradiation induced DNA damage foci (IRIFs) [49]. Similarly, ICP0 inhibits the recruitment of certain DNA repair proteins, such as 53BP1, but not others (for example γ-H2AX). These differences occur because IRIF formation is inhibited by ICP0 through inducing the degradation of RNF8 and RNF168 [49], which act downstream of the formation of γ-H2AX foci in the DNA damage response, but prior to the recruitment of 53BP1. These studies have also found evidence that recruitment of the DNA damage response proteins restricts the efficiency of ICP0 null mutant HSV-1 infection, thereby implicating this response in intrinsic resistance [45,49].

It is yet to be determined whether there are any connections between the mechanisms of recruitment of ND10 components and DNA damage response proteins to HSV-1 genome associated sites. The latter occurs in cells depleted of major ND10 components, and follows an analogous hierarchy of events to that of a DNA damage response in irradiated cells [45]. Furthermore, the accumulations of the DNA damage response proteins are spatially distinct from those of the recruited ND10 proteins [45] (Figure 1D). Therefore these recruitment events appear mechanistically and spatially distinct, but because IRIF formation has also been linked to the SUMO conjugation pathway [50,51], it is possible that there are some parallels between the two processes.

## 10. Conclusions

The recruitment of ND10 components to sites juxtaposed to HSV-1 genomes is a striking visual phenomenon. It occurs so rapidly that it can be seen before viral immediate-early proteins have reached detectable levels, and therefore it is one of the first indications that a cell has become infected. It is not surprising that nuclear entry of the HSV-1 genomes, comprising as it does 150 kbp of unchromatinized DNA, should trigger a storm of events involving multiple cellular responses and proteins. The recent work cited above demonstrates that one aspect of these events is involved in intrinsic resistance factors that aim to restrict expression of the foreign DNA. In retrospect, it is also not surprising that such a rapid response is mediated through individual protein molecules or small complexes, rather than substantial movement of either the viral genomes or pre-existing ND10—these are large structures that would be expected to have restricted mobility through the nucleoplasm.

Several major questions remain for future studies. What is the initial stimulus and what initially nucleates the recruited foci? What is the significance of the juxtaposition, rather than a precise co‑localization? What is the full catalogue of recruited proteins, and how do they link with transcriptional repression of the viral genome? What links are there between recruitment of these proteins and the assembly of the viral genome into the stably repressed chromatin configuration that is characteristic of cells that are latently or quiescently infected by HSV-1? These questions lie at the heart of HSV-1 biology, and are likely to stimulate much future research.
